# Supplementary figures and images for: Bioinspired activation strategies for Peano-HASEL artificial muscle
Source: PLoS One. 2025 Feb 6;20(2):e0318649. doi: 10.1371/journal.pone.0318649 (PMC11801529; doi:10.1371/journal.pone.0318649)

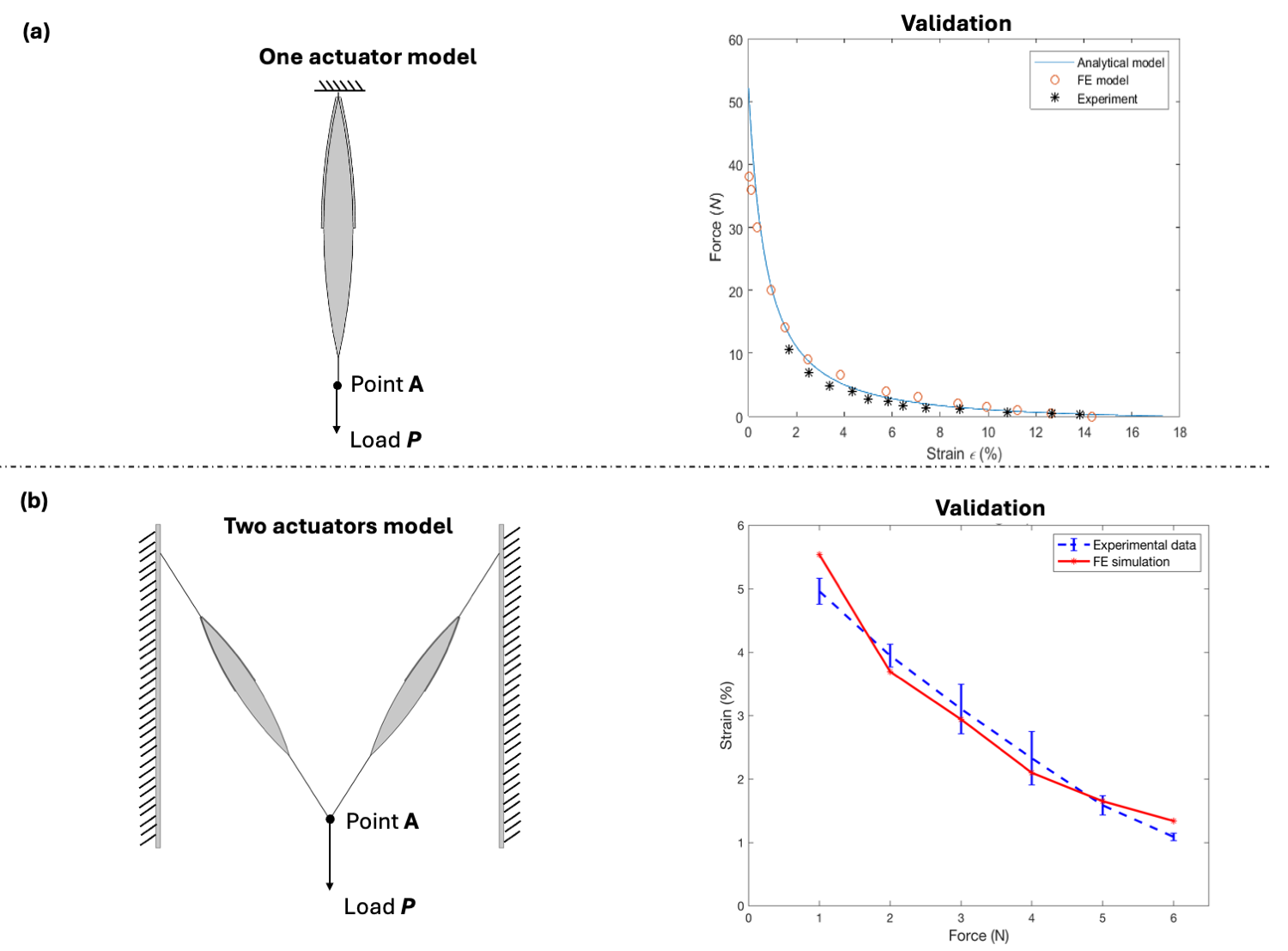

Supplement: S1 Fig — (TIF) [file pone.0318649.s001.tif]
