# Supplementary material for: Bioinspired activation strategies for Peano-HASEL artificial muscle
Source: PLoS One. 2025 Feb 6;20(2):e0318649. doi: 10.1371/journal.pone.0318649 (PMC11801529; doi:10.1371/journal.pone.0318649)
Supplement: S1 File — (DOCX) [file pone.0318649.s002.docx]

# S1. Validation of one and two actuators’ finite element model

S1 Fig shows the finite element models of one and two actuators and their force-length characteristics compared to experimental and analytical data from the literature. A boundary load P is applied at the bottom end of the actuator via point A and corresponding displacements are recorded. The force-strain relationships obtained from the finite element models are then compared to experimental data. The error between finite element simulations and experiments is less than 10%. This is strong evidence that the finite element simulations provide an accurate representation for PH actuator artificial muscle arrangements.

The main difference between the model used in the present study (i.e., four actuators) and our previous models (i.e., one and two actuators) is the number of actuators used. All the other factors like geometric parameters, material properties, the way to activate the actuators, and the way to connect actuators are the same. We have then made the assumption that as these other configurations based on the same parameters show good agreement with other data that it gives strong evidence that our model is accurate. Future work should begin experiments based on the promising activation strategies discovered in this paper.


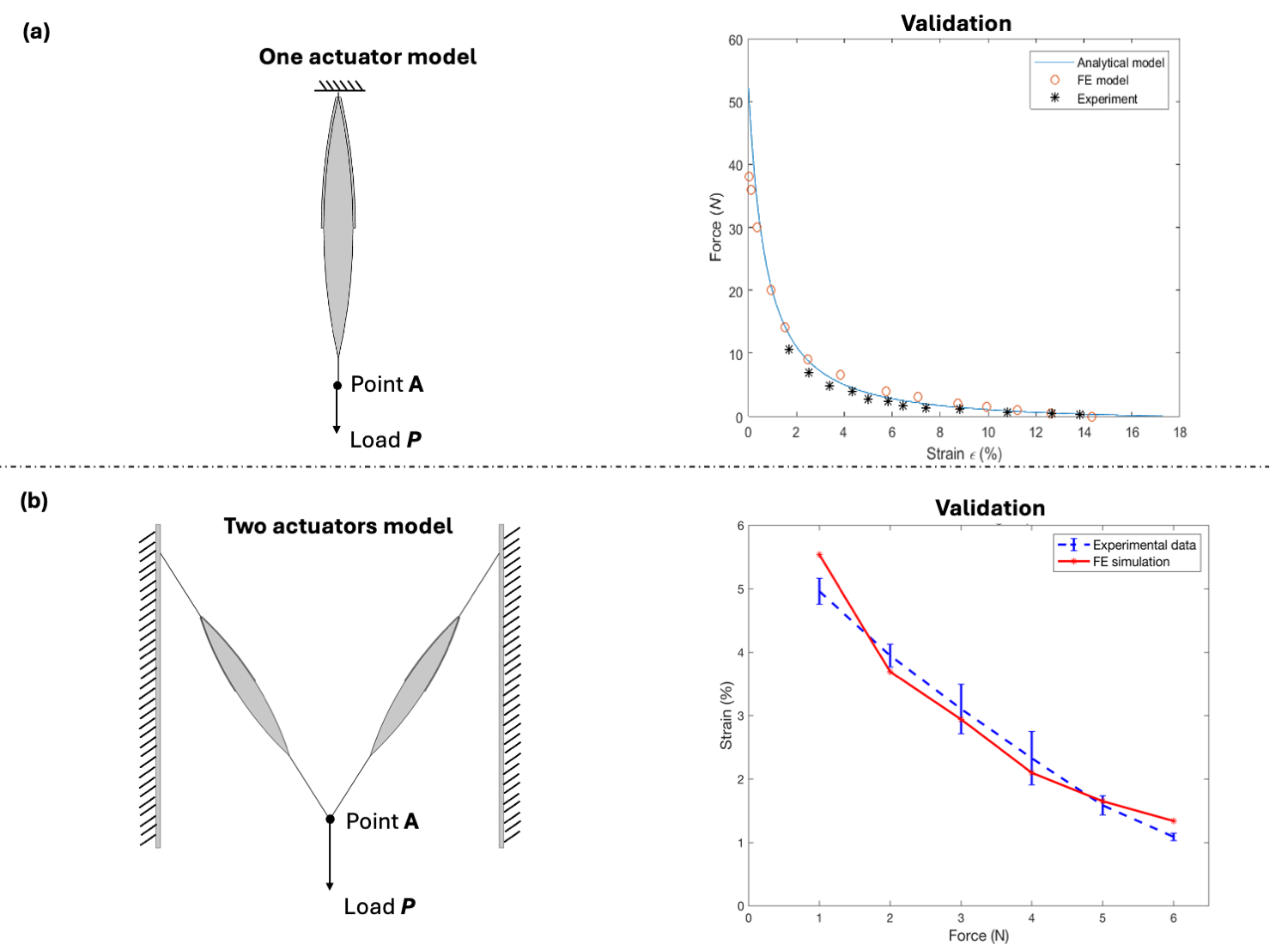


**S1 Fig. Validation of one and two actuators’ finite element model.** (a). One actuator model and validation process. A boundary load P ranging from 0 to 35 N is applied at point A and corresponding displacements are recorded. Then the force-strain relationship obtained from the model is compared with both analytical models and experimental data. (b). Two actuators model and validation process. A boundary load P ranging from 1 to 6 N is applied at point A and corresponding displacements are recorded. The force-strain relationship obtained from the model is compared with experimental data.
